# Treatment Response Prediction for Major Depressive Disorder Patients *via* Multivariate Pattern Analysis of Thalamic Features

**DOI:** 10.3389/fncom.2022.837093

**Published:** 2022-06-01

**Authors:** Hanxiaoran Li, Sutao Song, Donglin Wang, Danning Zhang, Zhonglin Tan, Zhenzhen Lian, Yan Wang, Xin Zhou, Chenyuan Pan, Yue Wu

**Affiliations:** ^1^Institutes of Psychological Sciences, College of Education, Hangzhou Normal University, Hangzhou, China; ^2^Center for Cognition and Brain Disorders, Hangzhou Normal University, Hangzhou, China; ^3^Zhejiang Key Laboratory for Research in Assessment of Cognitive Impairments, Hangzhou, China; ^4^School of Information Science and Engineering, Shandong Normal University, Jinan, China; ^5^Department of Psychiatry, The Affiliated Hospital, Hangzhou Normal University, Hangzhou, China; ^6^Shandong Mental Health Center, Shandong University, Jinan, Shandong, China; ^7^Department of Psychiatry, Hangzhou Seventh People’s Hospital, Hangzhou, China; ^8^Department of Translational Psychiatry Laboratory, Hangzhou Seventh People’s Hospital, Hangzhou, China

**Keywords:** major depressive disorder (MDD), thalamus, structural magnetic resonance imaging (sMRI), MVPA, treatment response prediction

## Abstract

Antidepressant treatment, as an important method in clinical practice, is not suitable for all major depressive disorder (MDD) patients. Although magnetic resonance imaging (MRI) studies have found thalamic abnormalities in MDD patients, it is not clear whether the features of the thalamus are suitable to serve as predictive aids for treatment responses at the individual level. Here, we tested the predictive value of gray matter density (GMD), gray matter volume (GMV), amplitude of low-frequency fluctuations (ALFF), and fractional ALFF (fALFF) of the thalamus using multivariate pattern analysis (MVPA). A total of 74 MDD patients and 44 healthy control (HC) subjects were recruited. Thirty-nine MDD patients and 35 HC subjects underwent scanning twice. Between the two scanning sessions, patients in the MDD group received selective serotonin reuptake inhibitor (SSRI) treatment for 3-month, and HC group did not receive any treatment. Gaussian process regression (GPR) was trained to predict the percentage decrease in the Hamilton Depression Scale (HAMD) score after treatment. The percentage decrease in HAMD score after SSRI treatment was predicted by building GPRs trained with baseline thalamic data. The results showed significant correlations between the true percentage of HAMD score decreases and predictions (*p* < 0.01, *r*^2^ = 0.11) in GPRs trained with GMD. We did not find significant correlations between the true percentage of HAMD score decreases and predictions in GMV (*p* = 0.16, *r*^2^ = 0.00), ALFF (*p* = 0.125, *r*^2^ = 0.00), and fALFF (*p* = 0.485, *r*^2^ = 0.10). Our results suggest that GMD of the thalamus has good potential as an aid in individualized treatment response predictions of MDD patients.

## Introduction

Major depressive disorder (MDD) is a common ailment that results in a colossal burden on families and society. Antidepressant treatment is an important method for providing relief for patients with MDD in clinical practice; however, only approximately 30% of MDD patients successfully achieve remission after 12 months of selective serotonin reuptake inhibitor (SSRI) therapy in the first level of the Sequenced Treatment Alternatives to Relieve Depression (STAR*D) trial (Papakostas et al., 2008). Some patients who failed to respond to antidepressant treatment chose to switch to another treatment later or continue the second therapy (Papakostas et al., 2008; Dunlop et al., 2017). The process of trial and error contributes to the consumption of time and money and even to delayed antidepressant treatment. One of the main reasons for such delays is the lack of predictive factors. Neuroimaging is a powerful tool to identify biomarkers of MDD as reliable predictors. Therefore, this study aimed to identify biomarkers and an individual-level method to aid in predicting the SSRI treatment responses of patients to give patients a more accurate medical plan, provide patients with more benefits, and save medical resources.

The thalamus might be a proper biomarker of MDD that could be used to predict patients’ antidepressant treatment responses. Our previous study and many other thalamic imaging studies have found thalamic abnormalities in patients with MDD, and these results show that the thalamus may play an important role in MDD (Li et al., 2021). Whether the thalamus is related to the prognosis of depression has also attracted the attention of researchers. A study demonstrated that pretreatment of patients with a smaller gray matter volume (GMV) in the thalamus was associated with a worse response to electroconvulsive therapy (Takamiya et al., 2019). This result has been confirmed by the latest review, in which thalamic volume reduction was shown to play a role in the outcome of a major depressive episode, possibly *via* its involvement in the pathophysiology of emotion (Batail et al., 2020). In addition to structural magnetic resonance imaging (MRI) studies, a study combining magnetoencephalography, positron emission tomography (PET), and repetitive transcranial magnetic stimulation (rTMS) affirmed impaired prefrontal-thalamic functional connections as a core deficit in treatment-resistant depression (TRD) (Li et al., 2013). In addition, patients with antidepressant TRD had increased right-thalamic fractional amplitude of low-frequency fluctuations (fALFF) values compared with patients without TRD (Yamamura et al., 2016). The abovementioned findings illustrate that the thalamus may be a potential biomarker of patients’ MDD treatment responses. Additionally, whether thalamic information could predict antidepressant treatment responses at the individual level is not clear. This study attempted to examine the performance of the structural and functional information of the thalamus to predict SSRI antidepressant medicine treatment responses at the individual level.

To predict individual antidepressant treatment responses based on thalamic information, multivariate pattern analysis (MVPA) techniques have been suggested as promising tools for predicting antidepressant treatment responses. In recent years, MVPA has been widely used to predict treatment responses at the individual level with high accuracy. Many studies have reported that machine learning models have good performance in identifying TRD patients (Liu et al., 2012; Redlich et al., 2016; Kautzky et al., 2017). The efficacy of the antidepressant medication fluoxetine and that of cognitive behavioral therapy were predicted based on brain structure information with 88.9% accuracy (Costafreda et al., 2009). With the application of the machine learning algorithm, an efficient prediction model with an accuracy of 75.0% for forecasting treatment outcomes could be generated, thus surpassing the predictive capabilities of clinical evaluation (Kautzky et al., 2018). These results indicated that prediction of TRD before undergoing a second round of antidepressant treatment could be feasible even in the absence of biomarker data (Nie et al., 2018). Most of the treatment predictions of MDD studies have focused on identifying TRD patients, although MDD continues to progress. Compared to classifiers, regression models can be performed at a continuous level, such as predicting Hamilton Depression Scale (HAMD) scores or score changes. Notably, Gaussian process regression (GPR) has been widely used in supervised machine learning due to its flexibility and inherent ability to describe uncertainty in function estimation (Hewing et al., 2019). To date, GPR has been used in mental disease research with good performance (Schmaal et al., 2018; Portugal et al., 2019; Peis et al., 2020).

To date, however, no researchers have used features of the thalamus as predictors of MDD to train machine learning models to explore individualized SSRI treatment response predictions of patients with MDD. Therefore, this study intends to explore the performance of resting-state functional MRI (rs-fMRI) [e.g., amplitude of low-frequency fluctuations (ALFF) and fALFF)] and sMRI [e.g., gray matter density (GMD) and GMV)] data of the thalamus to predict patients’ SSRI antidepressant medicine treatment responses by building GPR models. GPR was used in the present study to predict HAMD score changes for MDD patients after three months of SSRI antidepressant treatment.

## Materials and Methods

### Participants

In this study, 118 subjects were recruited, including 74 MDD patients (MDD group) and 44 healthy volunteers as the control group (HC group). Previous studies have shown that education level is a strong predictor of MDD and therefore should be strictly controlled in data analysis (Ladin, 2008; Chang-Quan et al., 2010; Park et al., 2013; Pearson et al., 2013; Johnson-Lawrence et al., 2019). Because it was difficult to match, education level was controlled as a covariate in the subsequent data processing by statistical techniques in the present study.

MDD patients (49 female patients and 25 male patients with an average age of 26.53 ± 8.56 years) were recruited from the Department of Psychiatry of the Seventh People’s Hospital of Hangzhou and the Department of Psychiatry of the Second People’s Hospital of Hangzhou. Study participants were the same as previously reported (Li et al., 2021). All enrolled patients met the following criteria: (1) met the International Classification of Diseases, 10th Revision (ICD-10) criteria for MDD; (2) had no history of medicine or physiotherapy for at least one month before recruitment or taking only SSRI antidepressants ≤1 week; (3) had a HAMD (Version: 24 Items, HAMD-24) total score ≥20; and (4) aged 18–65 years. There was no restriction on sex.

Healthy subjects (28 females and 16 males with an average age of 29.34 ± 12.42 years) were recruited from universities in Hangzhou and communities near hospitals by posters and internet announcements. The inclusion criteria were as follows: (1) did not meet the ICD-10 “depression episode” diagnostic criteria, had no family history of mental illness, and did not take any medication at least 1 month before recruitment; (2) HAMD-24 total score ≤ 8; and (3) aged 18–65 years.

**FIGURE 1:**
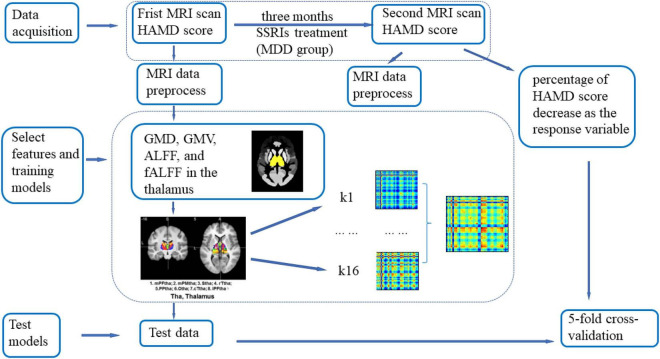
Illustration of the Gaussian process regression (GPR) procedure. Seventy-four major depressive disorder (MDD) patients and 44 healthy volunteers were included in the first magnetic resonance imaging (MRI) scan, and 39 MDD patients and 35 healthy control subjects were included in the second MRI scan. Between the two scans, participants in the MDD group received selective serotonin reuptake inhibitor (SSRI) treatment for 3 months, and individuals in the healthy control (HC) group did not receive any treatment. All datasets were preprocessed *via* DPABI_V3.1. GMV, GMD, ALFF, and fALFF values in the thalamus determined from the first scan were extracted as regression features. The percentage of Hamilton Depression Scale (HAMD) scores decreased as the response variable. The mask of the thalamus was first added to limit the brain region for analysis, and the Brainnetome Atlas, which divided the thalamus into 16 subregions, was added as a secondary mask. For every subregion, the signal in each voxel was extracted and concatenated as a feature vector. A vector was associated with the percentage of HAMD score decrease. Then, a linear kernel was built from the feature vectors for each region. The computed kernels were added to obtain a whole-thalamus linear kernel. The kernel and its associated percentages of HAMD score decrease were used to train the model and estimate the model parameters. The model could then give an associated predicted percentage of HAMD score decrease for new data. Fivefold cross-validation was used to evaluate the generalization performance of the models. A 1000-permutation test was performed to determine statistical significance, and cross-validation was repeated for each permutation.

Seventy-four participants, including 39 MDD patients (28 females and 11 males with an average age of 27.31 ± 8.36 years) and 35 HC subjects (24 females and 11 males with an average age of 28 ± 11.15 years), underwent scanning twice. Between the two scanning sessions, participants in the MDD group received SSRI (such as fluoxetine, paroxetine, sertraline, etc.) treatment for 3 months, and the participants in HC group did not receive any treatment (see Figure 1).

This study was approved by the Ethics Committee of the Institutes of Psychological Sciences, Hangzhou Normal University. All participants received written informed consent before participating in the study procedures.

### Magnetic Resonance Imaging Data Acquisition

Three-dimensional MR imaging was acquired using a GE 3T scanner (MR750, GE Medical Systems, Milwaukee, WI, United States) with a 32-channel radio frequency coil at the Center for Cognition and Brain Disorders (CCBD), Hangzhou Normal University (HZNU). MRI data acquisition were the same as previously reported (Li et al., 2021).

### Data Processing

#### Magnetic Resonance Imaging Data Preprocessing

All datasets were preprocessed *via* DPABI_V3.1 (a toolbox for Data Processing and Analysis for Brain Imaging) (Yan et al., 2016). More MRI data processing details were the same as previously reported (Li et al., 2021).

#### Features Used for Classification and Prediction

DPABI was used to make the whole-thalamus mask (Yan et al., 2016) and calculate the GMV, GMD, ALFF, and fALFF values. The participants’ GMV, GMD, ALFF, and fALFF values in the thalamus were extracted as regression and classification features. More information were the same as previously reported (Li et al., 2021).

#### Correlation Analysis

To detect the thalamic structural and functional indicators that are correlated with symptom relief (percentage of HAMD score decrease) in MDD patients, Pearson correlation analysis was conducted between the pretreatment thalamus indicators and the individual percentage of HAMD changes. We got the percentage of HAMD score decrease by subtracted the post-treatment score from the pre-treatment score to obtain the score difference, and divide the score difference by the score before treatment and multiply by 100.

#### Pattern Analysis

In this study, GPR was conducted for HAMD score predictions using the Pattern Recognition for Neuroimaging data Toolbox (PRoNTo)^1^ (Schrouff et al., 2013) (see Figure 1). GPR has been widely used in supervised machine learning due to its flexibility and inherent ability to describe uncertainty in function estimation (Hewing et al., 2019). Regression analysis has the potential to be used when examples (patterns) can be associated with a range of real values. The objective was to make continuous predictions. These values usually refer to demographic, clinical or behavioral data (such as age and HAMD scores) (Schrouff et al., 2013).

**FIGURE 2:**
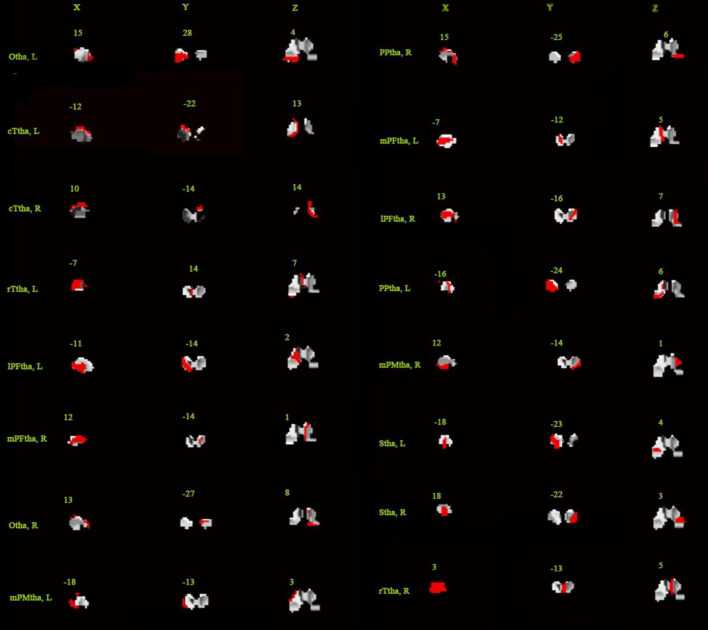
Subregions of the thalamus. mPFtha, medial prefrontal thalamus; mPMtha, premotor thalamus; Stha, sensory thalamus; rTtha, rostral temporal thalamus; PPtha, posterior parietal thalamus; Otha, occipital thalamus; cTtha, caudal temporal thalamus; lPFtha, lateral prefrontal thalamus; L: left; R: right. Adopted from Fan et al. (2016).

A mask of the thalamus was first added to limit the brain region for analysis, and the Brainnetome Atlas, which divided the thalamus into 16 subregions, was added as a secondary mask (see Figure 2; Fan et al., 2016). For every subregion, the signal in each voxel was extracted and concatenated as a feature vector. A vector was associated with the percentage of HAMD score decrease. Then, a linear kernel was built from the feature vectors for each region. The computed kernels were added to obtain a whole-thalamus linear kernel. The kernel and its associated percentage of HAMD score decrease were used to train the model and estimate the model parameters. The model could then give an associated predicted percentage of HAMD score decrease for new data (Schrouff et al., 2018). The correlation (r,$r=\frac{\sum_{n}\left(y_{n}-\mu_{y}\right)\,\left(f\,\left(x_{n}\right)-u_{f}\right)}{{\left\{\sum_{n}{\left(y_{n}-\mu_{y}\right)}^{2}\,\sum_{n}{\left(f\,\left(x_{n}\right)-\mu_{f}\right)}^{2}\right\}}^{\frac{1}{2}}}$) and the coefficient of determination (*r*^2^) between the targets (true percentage of HAMD score decrease) and the predictions (predicted percentage of HAMD score decrease) were used to evaluate the performance of the model. No parameters needed to be optimized during the model training. Fivefold cross-validation was used to evaluate the generalization performance of the models. A 1000-permutation test was performed to determine statistical significance, and cross-validation was repeated for each permutation.

## Results

### Sample Characteristics

Table 1 shows the demographic variables and clinical characteristics of participants in the two groups. Age (*Z* = −0.83, *p* = 0.410) and sex (χ^2^ = 0.08, *p* = 0.776) were well matched in participants in the MDD group (*Z* = −0.83, *p* = 0.410) and HC group, and there was no significant difference between these characteristics according to the Mann-Whitney test. Because the level of education was significantly higher in individuals in the HC group than in participants in the MDD group and may have had potential effects on the results, the level of education was used as an influencing factor for covariate analysis in all subsequent steps. HAMD-24 scores were also significantly higher in participants in the patient group than in individuals in the healthy group.

**TABLE 1:** Demographic and clinical characteristics of subjects.

Characteristic	MDD		HC		Statistic	
			
	First scan (*n* = 74)	Second scan (*n* = 39)	First scan (*n* = 44)	Second scan (*n* = 35)	First scan	Second scan
Age (years)	26.53 ± 8.56	27.31 ± 8.36	29.34 ± 12.42	28 ± 11.15	Z = −0.83	Z = −0.31
Sex, n (%)					χ^2^ = 0.08	χ^2^ = 0.92
Female	49 (66.22)	28 (71.79)	28 (63.64)	24 (68.57)		
Male	25 (33.78)	11 (28.21)	16 (36.36)	11 (31.43)		
Education level	4.68 ± 0.74	4.69 ± 0.73	5.43 ± 0.73	5.51 ± 0.61	χ^2^ = 39.24^***^	χ^2^ = 23.08***
HAMD-24 score	28.42 ± 6.22	12.72 ± 8.16	1.36 ± 1.37	1.14 ± 1.16	*t* = 36.01***	*t* = 7.24***

***p < 0.001. MDD, major depressive disorder group; HC, healthy control group; Education level, 1 (illiterate), 2 (primary school), 3 (junior high school), 4 (senior high school), 5 (college or university), 6 (master’s degree), 7 (doctorate); HAMD-24, Hamilton Depression Scale, Version: 24 Items.

### Correlation Between Antidepressant Treatment Response and Thalamus Magnetic Resonance Imaging Data Before Treatment

**FIGURE 3:**
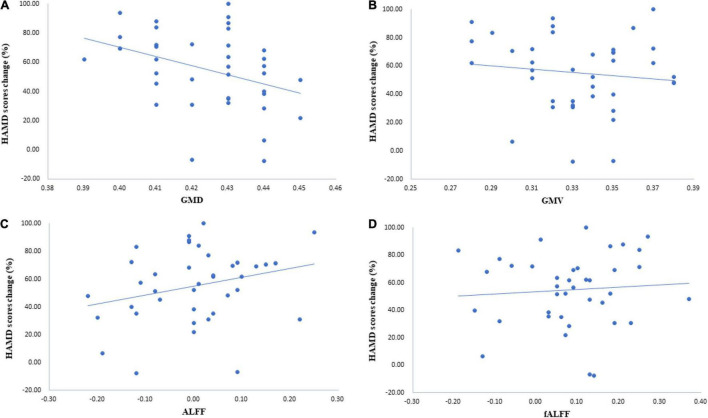
Correlation of thalamic characteristics and symptom relief. **(A)** The results illustrated that the correlation between gray matter density (GMD) and the percentage of the Hamilton Depression Scale (HAMD) score decrease was −0.329 (*p* = 0.041). **(B)** The correlation between gray matter volume (GMV)and the percentage of the HAMD score decrease was −0.132 (*p* = 0.424). **(C)** Results of correlation analysis with ALFF and the percentage of the HAMD score decrease (*r* = 0.27, *p* = 0.096). **(D)** Results of correlation analysis with fALFF and the percentage of the HAMD score decrease (*r* = 0.079, *p* = 0.631).

The regression analysis showed a strong negative correlation of thalamic GMD with the percentage of HAMD score decrease (*r* = −0.329, *p* = 0.041). In other words, lower pretreatment thalamus GMD was associated with a better clinical response (Figure 3A). This study did not find that GMV, ALFF, and fALFF of the thalamus were associated with the percentage of the HAMD score change (Figures 3B–D). The correlation between GMV and the percentage of the HAMD score decrease was -0.132 (*p* = 0.424), that between ALFF and the percentage of the HAMD score decrease was *r* = 0.27 (*p* = 0.096), and that between fALFF and the percentage of the HAMD score decrease was *r* = 0.079 (*p* = 0.631).

### Gaussian Process Regression Prediction of the Percentage of Hamilton Depression Scale Score Decrease for Participants in the Major Depressive Disorder Group After Treatment

#### The Change in Hamilton Depression Scale Score Predicted With Structural Features

**FIGURE 4:**
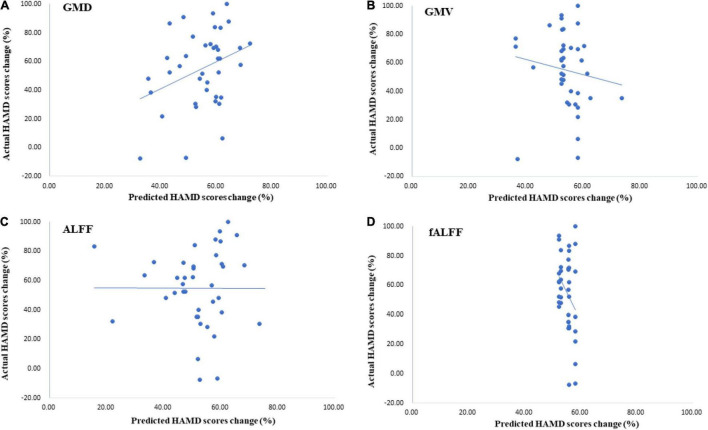
The results of Hamilton Depression Scale (HAMD) score predictions by Gaussian process regressions (GPRs) trained with thalamic ALFF, fALFF, GMD, and GMV data. **(A)** The GPR trained with GMDof the thalamus had a great performance in predicting the HAMD score 3 months later. Correlation was 0.34 (*p* = 0.01, *r*^2^ = 0.11). **(B)** The performance of GPR trained with GMV with the thalamus [correlation was −0.14 (*p* = 0.24, *r*^2^ = 0.02)]. **(C,D)** These two models had poor performance in HAMD score prediction after treatment.

Three months later, most MDD patients showed a decrease in HAMD scores. The percentage of change in the HAMD score was predicted by a GPR trained with baseline GMD and GMV data of the thalamus. Permutation tests showed a significant correlation between targets, i.e., the true percentage of the HAMD score decrease and predictions was 0.34 (*p* = 0.01, *r*^2^ = 0.11) in the GPR trained with GMD, and the correlation between targets and predictions was −0.14 (*p* = 0.24, *r*^2^ = 0.02) in the GPR trained with GMV (see Figures 4A,B).

#### The Change in Hamilton Depression Scale Scores Predicted With Functional Features

The results showed that the correlation between targets and predictions of the HAMD score change was -0.00 (*p* = 0.125, *r*^2^ = 0.00) with ALFF of the thalamus. For the GPR trained with fALFF data, the correlation between targets and predictions was −0.32 (*p* = 0.485, *r*^2^ = 0.10) (see Figures 4C,D).

### Group Analysis Results

#### Pretreatment Differences Between Major Depressive Disorder Patients and Healthy Controls

**FIGURE 5:**
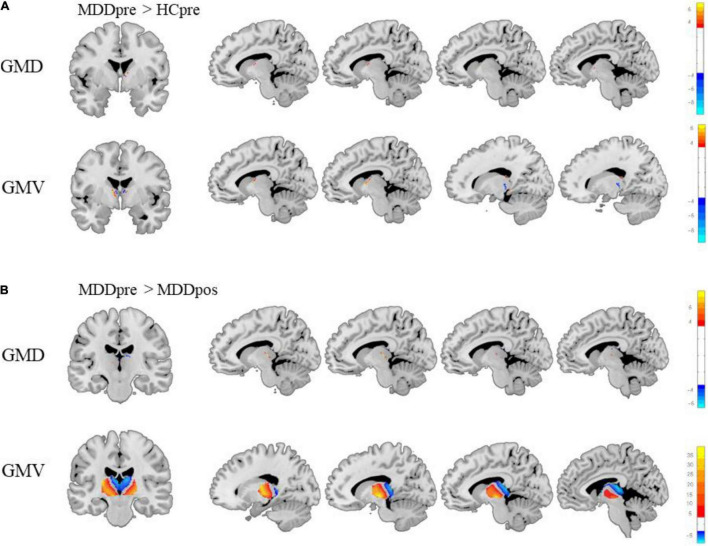
Differences between major depressive disorder (MDD) patients and healthy control (HC) subjects and changes in the thalamus of MDD patients after treatment. **(A)** There were significant differences between MDD patients and HC participants in thalamic gray matter density (GMD) and gray matter volume (GMV) before treatment. **(B)** The changes in the GMD and GMV of the thalamus in MDD patients after treatment (Gaussian random field-corrected, voxel *p*-value = 0.001, cluster *p*-value = 0.05).

Before treatment, both GMD and GMV of the thalamus in MDD participants were significantly different from those in HC subjects (Li et al., 2021). Compared to HC subjects, MDD patients showed higher GMD in the left rostral temporal thalamus and lower GMD in the right posterior parietal thalamus. Also, MDD patients showed higher GMV in the left lateral prefrontal thalamus, right posterior parietal thalamus, and right caudal temporal thalamus and lower GMV in the right medial prefrontal thalamus, right sensory thalamus, and left rostral temporal thalamus. After three months of treatment, there was no significant difference between MDD patients and HC participants in thalamic GMD and GMV. See Figure 5A.

No clusters showed significant differences between MDD patients and HCs in ALFF and fALFF of the thalamus.

#### Brain Changes in Major Depressive Disorder Patients After Treatment

This study showed a strong association between thalamic GMD and antidepressant effects. After 3 months of SSRI treatment, significant changes were observed in the GMD and GMV of the thalamus in MDD patients. The GMD was significantly increased in the rostral temporal thalamus (*t* = −4.36, *p* < 0.001), right rostral temporal thalamus (*t* = −6.32, *p* < 0.001), right occipital thalamus (*t* = −6.32, *p* < 0.001), and left medial prefrontal thalamus (*t* = −4.11, *p* < 0.001). The GMV was significantly increased in the bilateral ventromedial thalamus (*t* = −9.73, *p* < 0.001), and the GMV was significantly decreased in the bilateral dorsolateral thalamus (*t* = 39.91, *p* < 0.001). Moreover, after three months of treatment, there was no significant difference between participants in the MDD group and the HC group (see Figure 5B).

No clusters showed significant differences before treatment and after treatment in ALFF and fALFF of the thalamus in MDD patients.

## Discussion

To the best of our knowledge, this is the first study aimed at predicting MDD SSRI treatment responses by multivariate pattern analysis with sMRI of gray matter and rs-fMRI features in the thalamus. Correlation analysis and multivariate pattern analysis findings showed that GMD of the thalamus has a strong capability to predict the treatment responses of MDD patients. These findings indicate that the GMD of the thalamus may be a potential biomarker for MDD. In addition, the current results showed that after three months of SSRI treatment, abnormal thalamic changes in MDD patients altered the thalamic characteristics so that they were comparable to HC levels. This seems to indicate that SSRIs are beneficial to MDD patients, and the percentage decline in the HAMD score could provide evidence to prove this hypothesis.

Although medical treatment is the main antidepressant method, there are still many patients who are not sensitive to drug therapy, resulting in the delay of treatment. Although most studies exploring predictive analyzes in neuroimaging have been related to classification, regression analysis has aroused interest in the neuroscience community for its ability to allow researchers to decode continuous characteristics from neuroimaging data. It is important to foresee the antidepressant treatment response of MDD patients. Moreover, we trained the regression models with good performance. The correlation between the true percentage of HAMD score decrease and predictions was significant in the GPR trained with GMD. This result indicated that the GPR model could accurately predict the effect of antidepressant treatment. Many of the existing studies predicted treatment responses by classifying the patients into responders and non-responders; however, building classifiers is not the best model to predict treatment responses because of the continuity of the MDD patient’s recovery status. Compared to classifiers, GPR is more suitable for predicting treatment responses due to the ability to predict continuous data.

Our analysis of the longitudinal effects of antidepressants confirmed previous findings. Pertinently, the thalamic structure could be changed by antidepressant treatment. For instance, existing studies have shown a reduction in the thalamus after antidepressant treatment (Young et al., 2008). The change in MRI data proved objective evidence of the patient’s improvement. Moreover, our results indicated that the gray matter of the thalamus plays an important role in the treatment prediction of MDD. We successfully predicted the HAMD scores after three months of treatment with the pretreatment thalamus GMD value. Both previous studies and this study illustrated the potential of thalamic characteristics as biomarkers to predict the antidepressant treatment response, help determine the appropriate diagnosis and treatment plan and reduce the unnecessary waste of medical resources. In addition, the successful prediction of thalamic GMD may imply that this brain region may involve a pathophysiological mechanism of depression and may provide some clues for further research on the thalamus in emotion-related disorders.

The thalamus has been shown to be involved in the pathophysiological mechanism of MDD. Many existing studies have shown abnormalities of the thalamus in MDD patients. Compared with that of participants in the HC group, MDD patients had a smaller thalamus (Nugent et al., 2013). Other studies showed decreased left thalamus volume and contracted shape on ventral aspects of the left thalamus and decreased GMV of the right thalamus in MDD patients (Zhang et al., 2012; Zhao et al., 2014; Lu et al., 2016). In adolescents with MDD, GMV in the thalamus is inversely related to the severity of self-reported symptoms; furthermore, GMV in the thalamus decreases with age, while healthy adolescents show increases in thalamic GMV with age (Hagan et al., 2015). Notably, mental diseases may be related to thalamus abnormalities. A recent review of previous studies showed that the results from rodents indicate that thalamocortical circuits are candidates for controlling the activity of the default network, including task-suppression effects (Buckner and DiNicola, 2019). The dysregulation of thalamocortical circuits might also increase the risk of certain forms of mental illness (Buckner and DiNicola, 2019). Interestingly, MRI-related studies of MDD found that MDD patients had abnormalities in prefrontal, temporal, parietal, insula, occipital, and subcortical structures (Hao et al., 2019; Ma et al., 2019). The abovementioned brain areas are all related to thalamocortical circuits. If the gray matter of the thalamus, an important part of thalamocortical circuits, is abnormal, it may cause entire thalamocortical circuits to function abnormally, which may lead to MDD (Buckner and DiNicola, 2019). Therefore, the analysis of structural imaging data of the thalamus could separate MDD patients from healthy people.

There are some limitations to this study: (1) There was a significant difference in the level of education between the patient group and the control group, which may have impacted the results even though the education level was controlled. (2) The study sample was not very large, and this may have led to a deviation between the prediction results of the GPR and the true situation. By building a larger database upon which to base a predictive model, the variations observed among MDD patients could be more thoroughly incorporated, and in the future, this may result in models with better clinical utility (Patel et al., 2016). (3) Not all MDD patients were medicine-free subjects, and some of them were not experiencing their first depressive state. This may have influenced the results in the current study. These problems need to be addressed in future studies.

In conclusion, this was the first study to use a machine learning method to predict the HAMD score of MDD patients after three months of SSRI treatment with gray matter, ALFF, and fALFF data of the thalamus. MDD patients showed abnormal gray matter measurements, which could change in response to SSRI medicine. The GPRs trained with thalamic GMD data could predict the HAMD score of participants after three months of treatment and have been shown to have high discrimination accuracy by pattern analysis. Therefore, the results of this study suggest that GMD but not rs-fMRI information of the thalamus has good potential for SSRI treatment response predictions of MDD patients.

## Data Availability Statement

The raw data supporting the conclusions of this article will be made available by the authors, without undue reservation.

## Ethics Statement

The studies involving human participants were reviewed and approved by the Ethics Committee of the Institutes of Psychological Sciences, Hangzhou Normal University. The patients/participants provided their written informed consent to participate in this study.

## Author Contributions

DW originated the study and revised the manuscript. SS and DZ supervised the data processing and revised the manuscript. HL conducted the statistical analyses and drafted the manuscript. ZT, ZL, YaW, XZ, YuW, and CP collected the relevant data. All authors have read and agreed with the contents of the manuscript, contributed to the article, and approved the submitted version.

## Conflict of Interest

The authors declare that the research was conducted in the absence of any commercial or financial relationships that could be construed as a potential conflict of interest.

## Publisher’s Note

All claims expressed in this article are solely those of the authors and do not necessarily represent those of their affiliated organizations, or those of the publisher, the editors and the reviewers. Any product that may be evaluated in this article, or claim that may be made by its manufacturer, is not guaranteed or endorsed by the publisher.

## Footnotes

1
http://www.mlnl.cs.ucl.ac.uk/pronto
